# Chromatin architecture reveals cell type-specific target genes for kidney disease risk variants

**DOI:** 10.1186/s12915-021-00977-7

**Published:** 2021-02-24

**Authors:** Aiping Duan, Hong Wang, Yan Zhu, Qi Wang, Jing Zhang, Qing Hou, Yuexian Xing, Jinsong Shi, Jinhua Hou, Zhaohui Qin, Zhaohong Chen, Zhihong Liu, Jingping Yang

**Affiliations:** 1 National Clinical Research Center for Kidney Disease, Jinling Hospital, Medical School of Nanjing University, Nanjing, 210002 Jiangsu China; 2 Medical School of Nanjing University, Nanjing, 210093 Jiangsu China; 3 Department of Biostatistics and Bioinformatics, Rollins School of Public Health, Emory University, 1518 Clifton Road N.E, Atlanta, GA 30322 USA

**Keywords:** Chromatin organization, Epigenetic landscape, Regulatory element, Disease-associated variant

## Abstract

**Background:**

Cell type-specific transcriptional programming results from the combinatorial interplay between the repertoire of active regulatory elements. Disease-associated variants disrupt such programming, leading to altered expression of downstream regulated genes and the onset of pathological states. However, due to the non-linear regulatory properties of non-coding elements such as enhancers, which can activate transcription at long distances and in a non-directional way, the identification of causal variants and their target genes remains challenging. Here, we provide a multi-omics analysis to identify regulatory elements associated with functional kidney disease variants, and downstream regulated genes.

**Results:**

In order to understand the genetic risk of kidney diseases, we generated a comprehensive dataset of the chromatin landscape of human kidney tubule cells, including transcription-centered 3D chromatin organization, histone modifications distribution and transcriptome with HiChIP, ChIP-seq and RNA-seq. We identified genome-wide functional elements and thousands of interactions between the distal elements and target genes. The results revealed that risk variants for renal tumor and chronic kidney disease were enriched in kidney tubule cells. We further pinpointed the target genes for the variants and validated two target genes by CRISPR/Cas9 genome editing techniques in zebrafish, demonstrating that SLC34A1 and MTX1 were indispensable genes to maintain kidney function.

**Conclusions:**

Our results provide a valuable multi-omics resource on the chromatin landscape of human kidney tubule cells and establish a bioinformatic pipeline in dissecting functions of kidney disease-associated variants based on cell type-specific epigenome.

**Supplementary Information:**

The online version contains supplementary material available at 10.1186/s12915-021-00977-7.

## Background

Over the past decade, genome-wide association studies (GWAS) have successfully identified tens of thousands of genetic variants associated with a wide variety of human diseases, including kidney diseases [1, 2]. However, the identification of the causal variants and the search for their target genes that underlie these variants are extremely challenging. To address this, it is important that we incorporate additional information to assist the identification process. Among many options, the rapid growth of the epigenomic information provides a valuable resource for functional variant annotation [3, 4].

A large proportion of disease-associated variants are located in the non-coding region of the genome [5, 6]. It has been reported that functional non-coding regions are sensitive to digestion by enzymes like DNase or Tn5 [7, 8]. Furthermore, histone modifications have been linked to specific functional annotation of non-coding regions [9]. As examples, active promoter regions are often found to be marked by H3K4me3 and transcriptional activators like enhancers are often found to be marked by H3K27ac [10]. Jung et al. have combined ATAC-Seq and H3K27Ac ChIP-seq to identify potential enhancers for AQP2 in collecting duct principal cells (mpkCCD) [11]. Compared to causal variant identification, searching for the target genes of these variants is even more challenging. Regulatory elements such as enhancers can regulate their target genes regardless of distance and direction [12]; hence, it is unreliable to predict their target genes by genomic proximity alone. Instead, it has been demonstrated that physical contact via chromatin folding is necessary for gene activation by enhancer [13, 14]. Investigations of the contact through chromatin conformation capture technologies have enabled a systematic understanding the molecular targets deregulated in distinct diseases and traits [15, 16]. From the above, we believe that epigenomic features can help distinguish the causal variants from the others and facilitate the identification of the target genes for regulatory variants.

The epigenomic features are tissue and cell type-specific [17]. Existing studies only survey a limited number of epigenomic features on selected human tissues and cell types. For kidney, the Roadmap Epigenome Mapping Consortium (REMC) has collected various histone modifications for the whole kidney [18], but no matching chromatin interaction map has been generated in whole kidney. Furthermore, some studies generated chromatin interaction maps in kidney, but there is no mapping histone modification profile for any specific kidney cell types such as tubule cells. In a recent study, Brandt et al. applied 4C-seq (circular chromatin conformation capture) on a group of kidney disease-associated variants to search for their target genes in kidney cells [12]. Since 4C-seq is not designed for genome-wide conformation capture, the information is limited to the selected loci [19]. In another study, Sieber et al. combined DNase map on glomeruli cell culture and Hi-C (high-throughput chromosome conformation capture) on freshly isolated glomeruli to shed light on glomeruli genome function [20]. As DNase map does not provide annotation that can distinguish different types of regulatory elements such as enhancers or promoters that combined histone modifications are able to provide, and the Hi-C has yet to achieve sufficient resolution to delineate transcription-related chromatin interaction [19], more experimental data are needed in order to build a refined epigenetic landscape to achieve variant annotation in the kidney [21].

Here we incorporated high-resolution and comprehensive one-dimension (1D) to three-dimension (3D) epigenetic landscapes in kidney tubular epithelial cell model HK2 cells which retained functional characteristics of proximal tubules [22, 23] to annotate variants identified by GWAS. We captured the chromatin interaction profile using HiChIP (in situ Hi-C followed by chromatin immunoprecipitation) which is a protein selected chromosome conformation capture technology. Compared to Hi-C at comparable sequencing depth, HiChIP would increase the resolution by 10 fold therefore enables accurate detection of chromatin interactions [24, 25]. In parallel with the high-resolution contact map, we integrated multi-layer histone modifications annotation such that different types of functional elements could be recognized. Using this system, we established a regulatory network and applied it to explore the role of GWAS hits in kidney diseases. Our analyses discovered enrichment of causal variants for renal cell carcinoma (RCC) and chronic kidney disease (CKD) in kidney tubular cells. We established a pipeline from epigenomic landscape generation to GWAS hits annotation and provided a comprehensive resource for further investigation of any genomic locus of interest in the kidney tubular cells.

## Results

### Identification of genome-wide cell type-specific regulatory elements

**Fig. 1:**
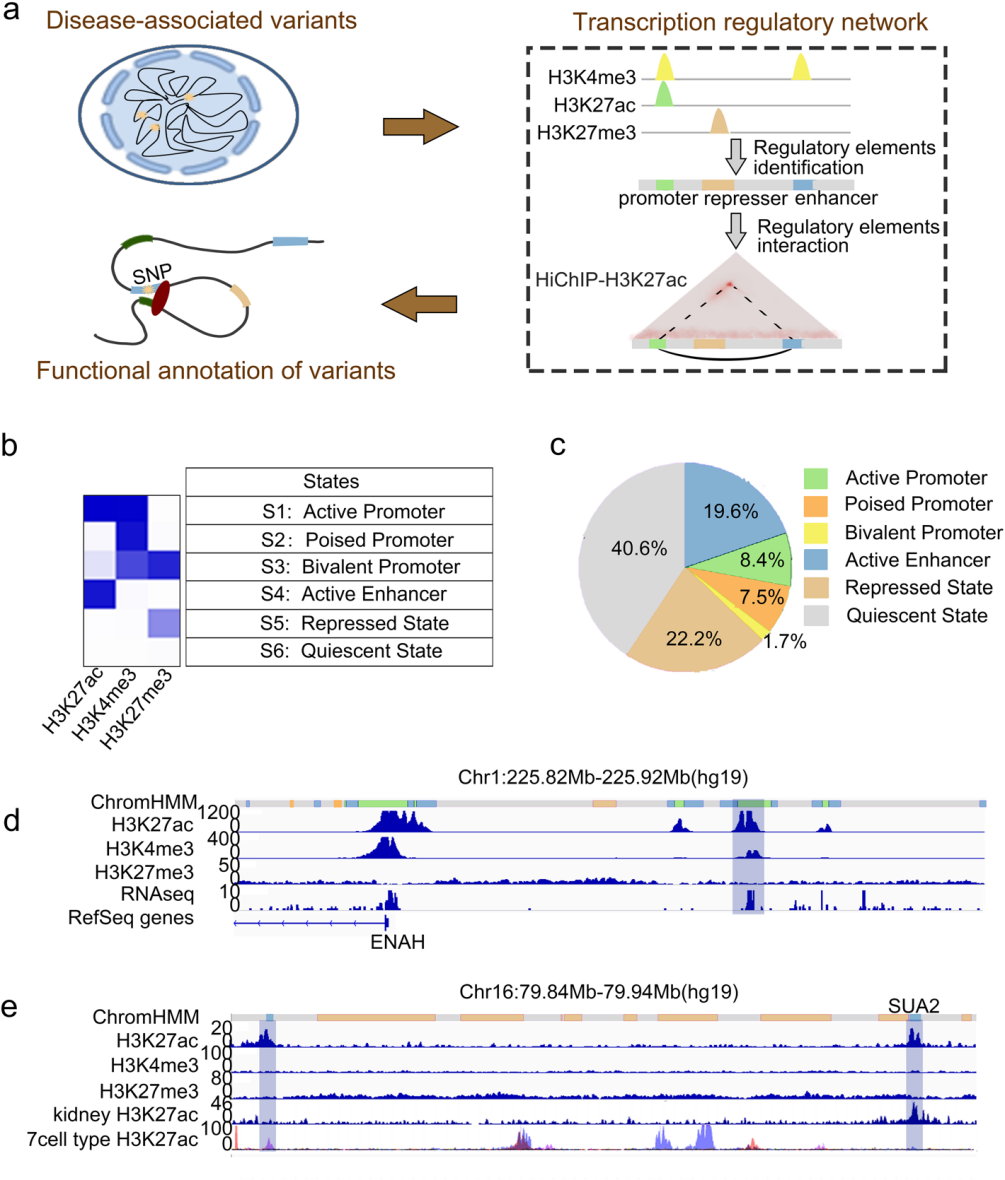
Identification of regulatory elements in tubular cells. **a** Schematic of strategy to functionally annotate disease-associated variants with 1D and 3D epigenetic landscapes. **b** ChromHMM-based chromatin states in tubular cells. Each row corresponds to a different state, and each column corresponds to a different mark for three histone modifications (H3K27ac, H3K4me3, H3K27me3). The blue color represents the probability for each mark in the state. **c** Proportion of the regions for each state in genome. **d** Segmentation state, H3K27ac, H3K4me3, H3K27me3 and RNA-seq tracks on chromosome 1 (225.82–225.92 Mb). Blue block highlights a novel promoter not defined by Refseq. **e** Segmentation state, H3K27ac, H3K4me3, H3K27me3, kidney H3K27ac and 7-cell type H3K27ac profiles on chromosome 16 (79.84–79.94 Mb). Blue blocks highlight enhancers including SUA2 and a novel enhancer. Kidney H3K27ac and 7-cell type H3K27ac profiles were from ENCODE

In order to annotate kidney disease-associated variants, we need to establish a cell type-specific transcription regulatory network (Fig. 1a). As kidney tubules play important roles in kidney diseases [22, 23], we firstly identified functional elements in kidney tubular cells. To achieve this, we experimentally constructed multiple histone modification profiles including H3K4me3, H3K27ac and H3K27me3 (Additional file 1: Table S1, Additional file 1: Fig. S1) to call cell type-specific functional elements. When compared to histone modifications in whole kidney [26], it was revealed that the data retained characteristics of proximal tubules in kidney, without signal at non-tubular feature genes (Additional file 1: Fig. S2). We then applied ChromHMM on these histone modification profiles jointly and discovered six chromatin categories including active promoter, poised promoter, bivalent promoter, active enhancer, repressed state and quiescent state (Fig. 1b). In total, we identified 51,569 promoters in kidney tubular cells representing about 17.6% of the genome (Fig. 1c), including Refseq-annotated promoters as well as novel promoters that have not been annotated in current genome annotation (Fig. 1d). Currently, in kidney cells including tubular cells, enhancers are poorly annotated. Our histone landscape predicted 57,345 active enhancers with H3K27ac but lacking H3K4me3, representing 19.6% of the genome (Fig. 1c). Previously reported enhancer SUA2 [27] and enhancer in DAB2 loci [28] were confirmed by the histone landscape we derived (Fig. 1e and Additional file 1: Fig. S3). The majority of the enhancers we discovered have never been annotated before.

**Fig. 2:**
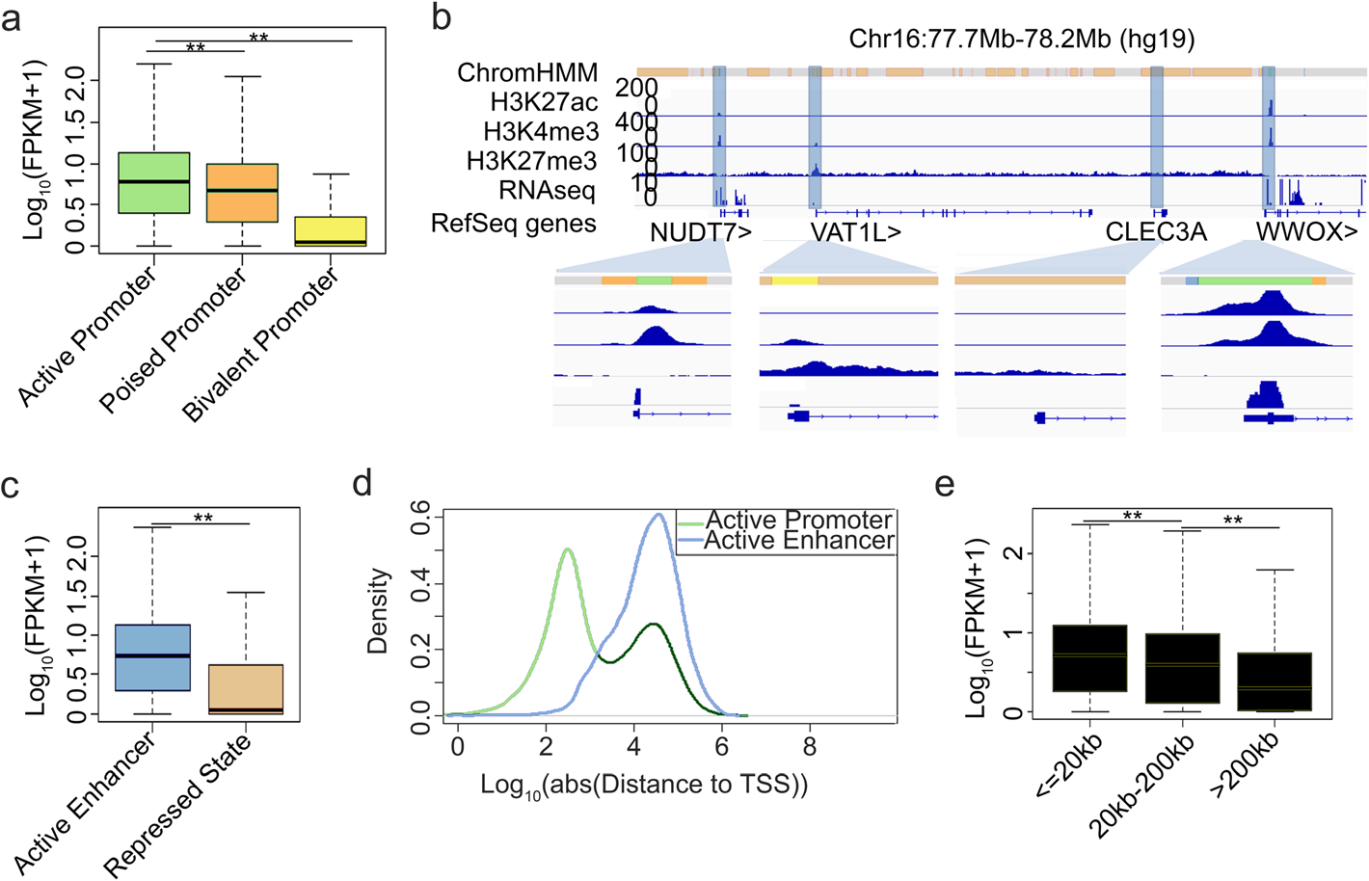
Effect of regulatory elements on gene transcription regulation. **a** Boxplots of transcription for genes associated with active promoter, poised promoter, and bivalent promoter. ** represents *p* value < 0.01. **b** Segmentation state, H3K27ac, H3K4me3, H3K27me3, and RNA-seq profiles on chromosome 16: 77.7–78.2 Mb. Promoters of NUDT7, VAT1L, CLEC3A, and WWOX are highlighted (blue shading) and enlarged. **c** Boxplots of transcription for genes associated with active enhancer and repressed state. ** represents *p* value < 0.01. **d** Distribution of distance between regulatory elements and TSS of their nearest genes. **e** Boxplots of transcription for genes by active enhancer with corresponding distance. ** represents *p* value < 0.01

To determine the functional potential of these predicted regulatory elements, we next examined the expression level of the genes targeted by these regulatory elements. We found that the expression of genes associated with active promoter was significantly higher than the other genes (Fig. 2a). As an example, in a 500-kb region on chr16, four promoters were predicted with diverse states. NUDT7 and WWOX, two genes found to be associated with active promoters, are highly expressed, whereas VAT1L and CLEC3A, two genes associated with either bivalent promoter or repressed state, are not expressed, at both transcript level (Fig. 2b) and protein level (Additional file 1: Fig. S4). Our results revealed that the state of promoters directly regulated transcription activity of their associated genes.

For non-promoter elements, we examined their effect on the nearest gene. We found that the expression of genes associated with active enhancer is much higher than that of genes associated with repressive elements (Fig. 2c), confirming the activation potential of these enhancers. As the identified enhancers are distal elements to genes with an average distance of 54.7 kb (Fig. 2d), we further grouped the enhancers by the distance to their nearest genes. The result revealed that the regulatory potential of active enhancers declined as the distance increased. For enhancers more than 200 kb away from their nearest genes, the average expression level of their nearest genes decreased by more than 2.5 fold (Fig. 2e), suggesting that their nearest genes may not be the real targets for those distal enhancers. Taken together, we established a cell type-specific 1D regulatory landscape with histone modification patterns genome-wide and identified functional elements for the transcriptome, but the true target genes of these elements remain elusive especially for those distal enhancers.

### Establishment of regulatory network in 3D genome

**Fig. 3:**
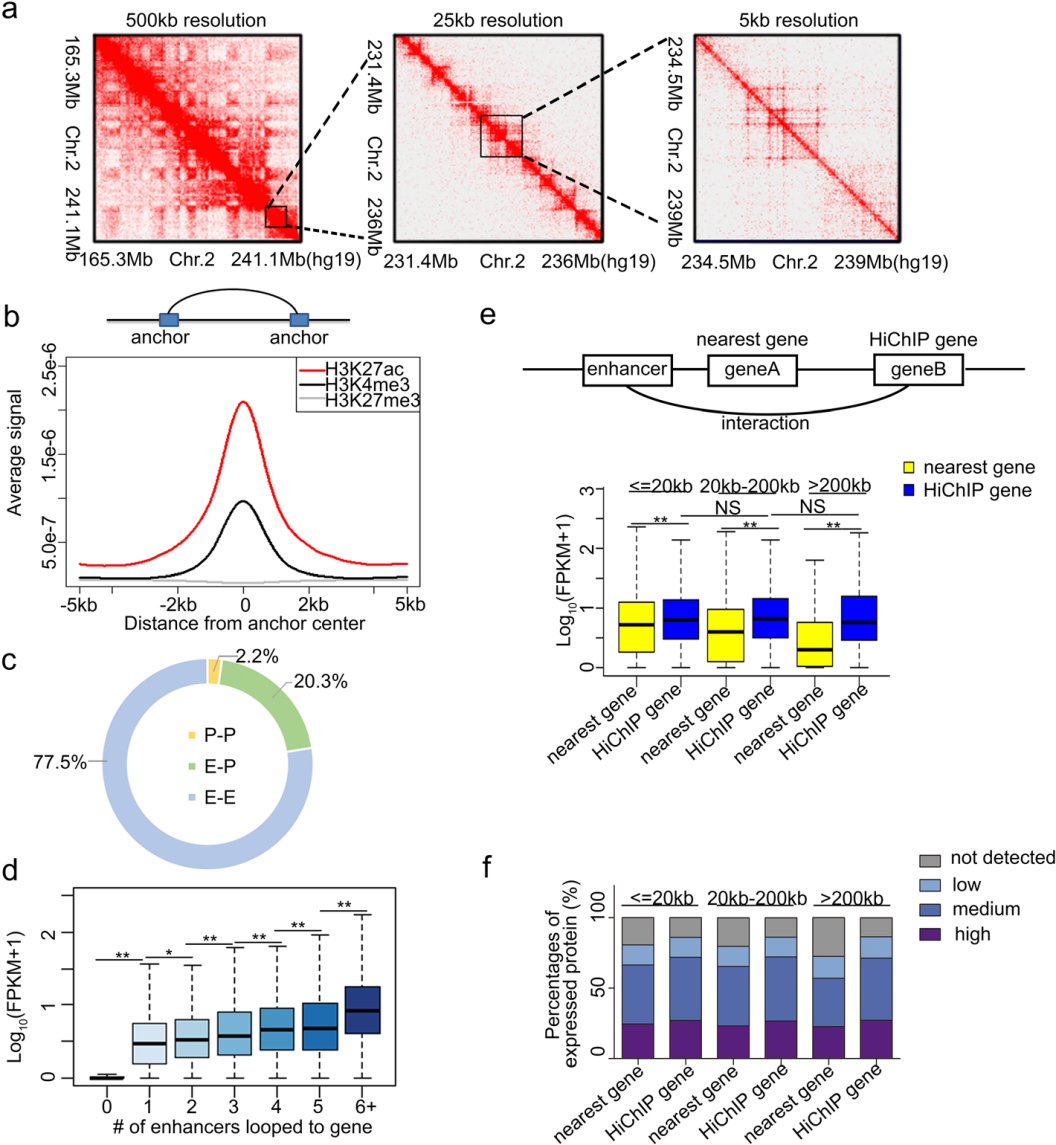
Chromatin interaction map in tubular cells. **a** Knight–Ruiz (KR) matrix-balanced interaction maps for tubular cells at 500-kb, 25-kb, and 5-kb resolution. **b** Upper: Schematic diagram of definition of the anchor. Bottom: three ChIP-seq signals around chromatin anchor midpoints. **c** Proportion of interaction types. **d** Transcription levels for genes interacted with different numbers enhancers. ** represents *p* value < 0.01. * represents *p* value < 0.05. **e** Upper: Schematic diagram of definition of nearest gene and HiChIP gene. Bottom: Transcription levels for nearest or HiChIP genes for the same enhancer. ** represents *p* value < 0.01. **f** The percentages of protein expression for nearest and HiChIP genes in kidney tubule from Human Protein atlas

In order to identify the target genes for distal elements, we next developed a high-resolution chromatin contact map (Fig. 3a, Additional file 1: Table S2, Additional file 1: Fig. S5). In total, 239,716 interactions were detected throughout the genome. Generated using HiChIP with H3K27ac, the contact map is transcription-centered. The anchors of the interactions were enriched for active histone markers (Fig. 3b). Comparing the location of these anchors to the identified regulatory elements revealed that 77.5%, 2.2%, and 20.3% of the interactions are between enhancers (e-e), between promoters (p-p), and between enhancers and promoters (e-p) respectively (Fig. 3c). Since enhancers could increase transcriptional bursting of their target genes, we then examined whether contacting with enhancers increased the expression of a gene. Compared to genes contacted with no enhancer, the genes interacted with enhancers displayed significantly higher expression levels. Additionally, we found that contact with more enhancers further increased the expression (Fig. 3d), confirming that enhancer interaction is important for transcription activation.

The potential target genes of identified active enhancer are defined either as the nearest genes or the genes interacted with active enhancer in the 3D interaction map (HiChIP gene) (Fig. 3e). For an active enhancer, we found that the HiChIP gene showed more active expression than the nearest gene, especially for those interacted with distal enhancers (> 200 kb). Additionally, as the 3D chromatin interaction has little to do with linear genomic distance, the activation potential for interacted genes did not decline as that for nearest genes (Fig. 3e). Consistently, when we examined the expression of identified target genes in kidney tubule in vivo, we found that HiChIP genes expressed more frequently than nearest genes did and the proportion of expressed genes did not decrease with the distance (Fig. 3f). The results indicated that the chromatin interaction map generated by HiChIP reflect the regulatory network more accurately, especially for distal enhancers. The interacted genes are more likely to be the real target genes in transcription regulation for these enhancer elements.

### Functional annotation of causal variants associated with kidney related traits

**Fig. 4:**
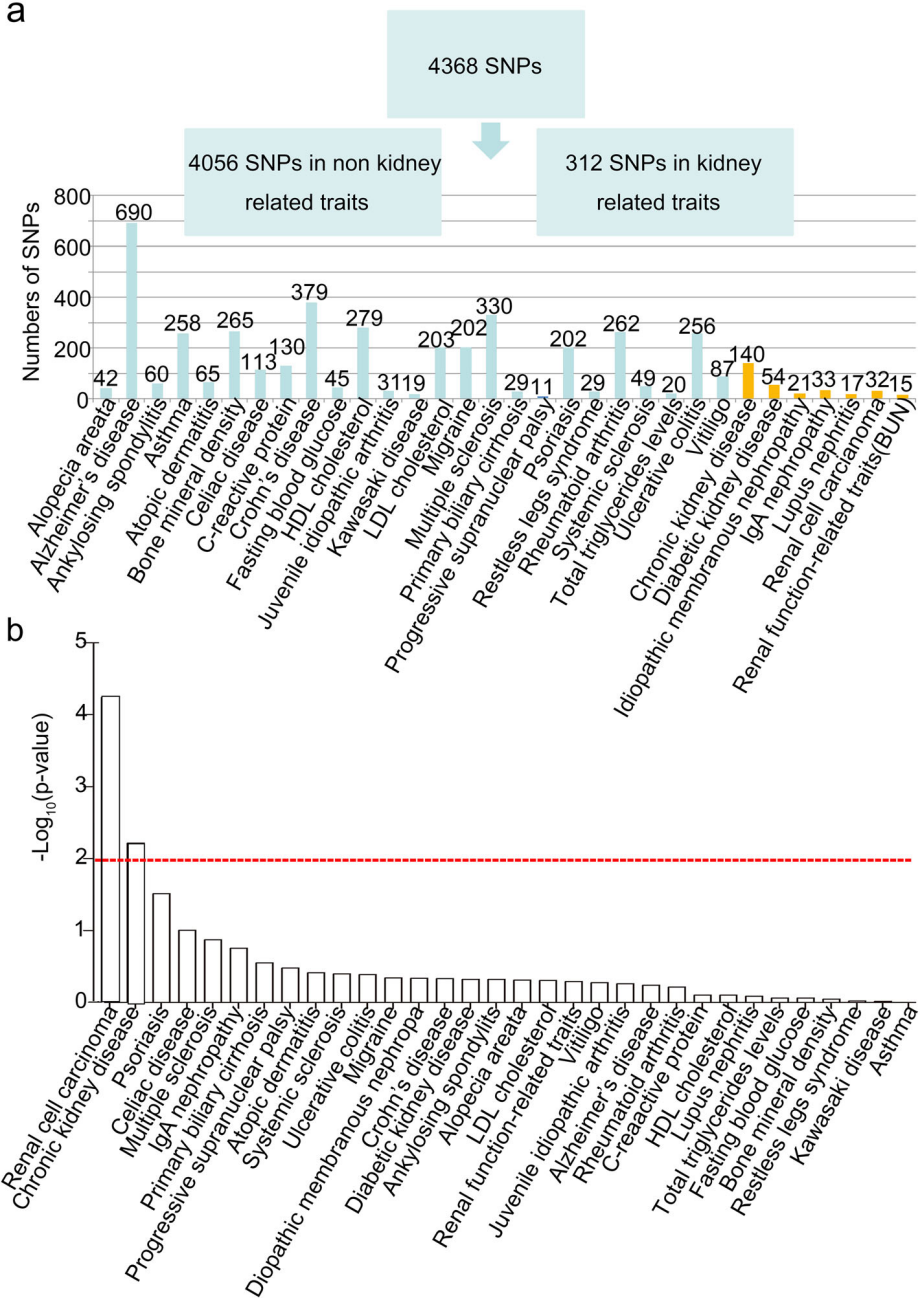
Discovery of functional SNPs for kidney related traits. **a** Number of GWAS SNPs for kidney and non-kidney-related traits. *Y*-axis represents the number of SNPs. Cyan bars represent non-kidney-related traits, and yellow bars represent kidney-related traits. **b** The enrichment level of GWAS SNPs at active enhancer for different traits. Red line: *p* value = 0.01

The identified regulatory elements and chromatin interaction map enable us to interpret the function of non-coding variants, which is impractical to assay by standard approaches such as eQTL (expression quantitative trait loci) mapping without very large sample sizes. We collected disease-associated SNPs from GWAS catalog (downloaded 30 September 2018) and CKDGen consortium (downloaded 24 April 2019), including 312 SNPs for kidney-related traits (Fig. 4a). By assessing overlap between the GWAS hits and our identified cell type-specific regulatory elements, we found a group of SNPs that co-localize with various types of regulatory elements. Among all kidney-related trait-associated GWAS SNPs, 12% overlapped with identified promoters and 17% overlapped with identified active enhancer in tubular cells (Additional file 1: Table S3 and Additional file 2: Table S5). Through bootstrap simulation using all disease-associated SNPs as control, the results revealed significant overlap between GWAS hits for renal cell carcinoma (RCC) (*p* value = 5.71e^− 5^) or CKD (*p* value = 0.006) and identified active enhancers (Fig. 4b and Additional file 1: Fig. S6a), but overlap with all every other type of regulatory elements is insignificant (Additional file 1: Fig. S6b). This discovery was confirmed by the report that RCC was likely derived from kidney proximal tubule cells [29] and emphasized the importance for cell type-specific annotation for GWAS hits.

**Fig. 5:**
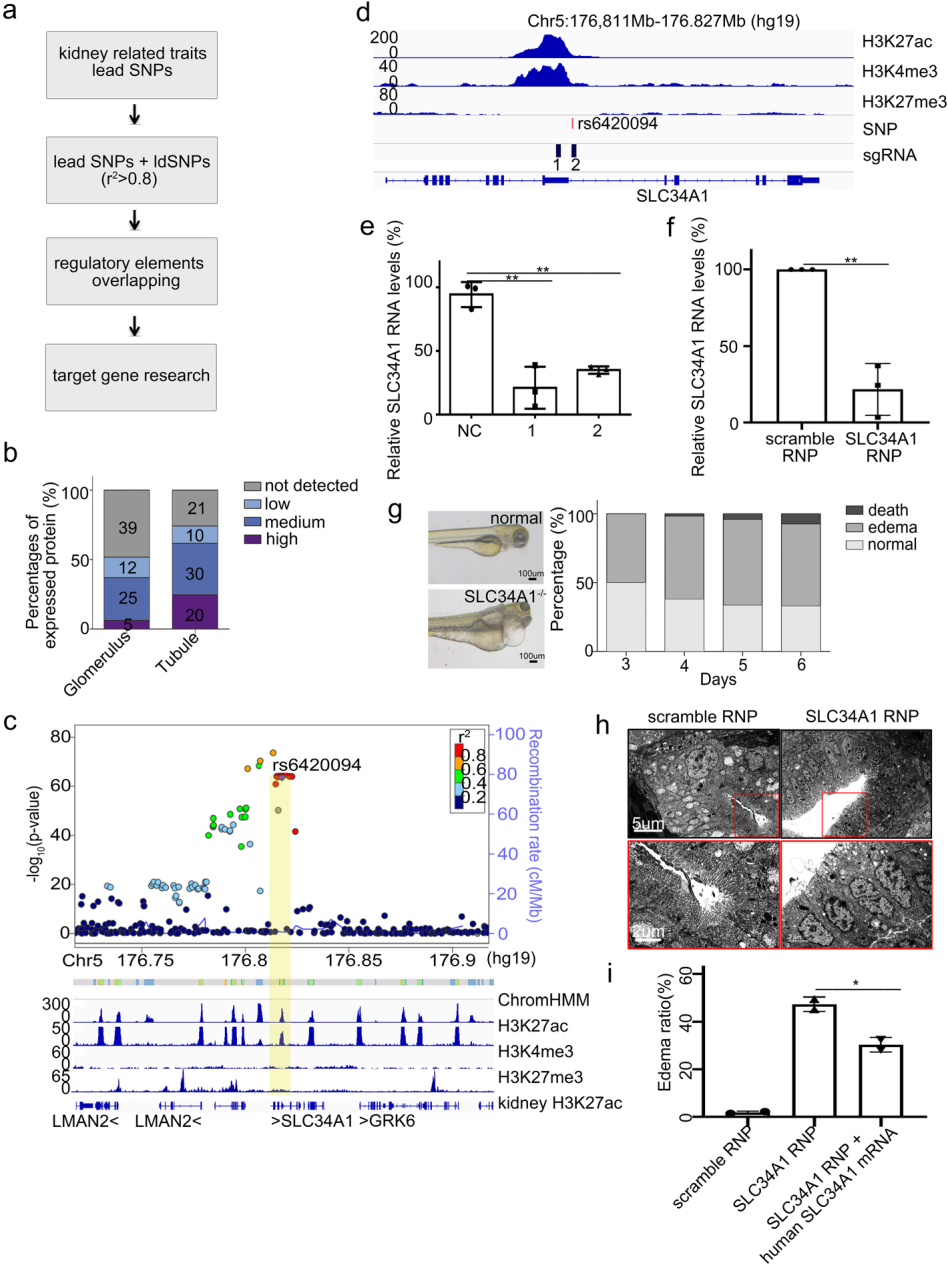
Identification of target genes for trait-associated SNPs in promoters. **a** Flowchart for the identification of target genes for kidney related traits. **b** The expression of target genes for SNPs overlapping with promoters in glomeruli and tubules. The expression categories are characterized based on immunochemistry results by Human Protein Atlas (HPA). The numbers represent the number of genes in each category. **c** Top: LocusZoom plot from CKD GWAS around the region of rs6420094. Bottom: epigenomic profile including ChromHMM, histone modifications in tubular cells, histone modification in kidney, and gene annotation track. Yellow bar highlights rs6420094. **d** Tracks of histone modifications, locations of risk SNP rs6420094 and sgRNAs designed to inactivate the element. **e** qPCR of SLC34A1 transcription with or without CRISPRi of the element. ** represents *p* value < 0.01. Each group was repeated three times. **f** qPCR of SLC34A1 transcription in zebrafish with RNP (sgRNA and CRISPR/Cas9 protein complex) of scramble, SLC34A1 sgRNA. Each group was repeated three times. **g** Left: representative figures show edema of SLC34A1^−/−^ zebrafish. Right: edema ratio of SLC34A1^−/−^ zebrafish at different days. **h** Electron microscope of kidney from zebrafish with scramble, SLC34A1 RNP. The red boxes (top) show increase space between tubules, and they are enlarged (bottom) to show the shed off of brush-border fragment. **i** Overexpression of human SLC34A1 mRNA partially rescue the edema. Each group were examined on two batches

To further investigate the underlying role of disease-associated SNPs, we next searched for target genes of these functional SNPs in our chromatin contact map. We included lead SNPs as well as SNPs in linkage disequilibrium (*r*^2^ > 0.8, referred to as ldSNPs) for target gene hunting (Fig. 5a). Among the 5745 SNPs, 333 located at promoters (Additional file 3: Table S6 and Additional file 4: Table S7). For the 101 genes with SNPs at their promoters, 74.07% expressed at protein level in kidney tubules (Fig. 5b). The expression frequency was significantly higher than that in glomeruli (*p* = 0.0054). For example, rs6420094 that associated with CKD was located in the active promoter of SLC34A1 in our landscape (Fig. 5c). The expression of SLC34A1 was tubule specific, and its expression showed downregulation in chronic kidney disease (Additional file 1: Fig. S7a and Additional file 1: Fig. S7b). To validate the regulatory role of rs6420094 element on SLC34A1 transcription, we designed sgRNAs and use CRISPR interference (CRISPRi) [30] to inactivate the rs6420094 element. The results showed that silencing of the rs6420094 element reduced SLC34A1 expression significantly (Fig. 5d and Fig. 5e). To test the role of the target gene in kidney function, we used CRISPR/Cas9 to knock out SLC34A1 in zebrafish (Fig. 5f). The results showed that SLC34A1 knockout lead to kidney function defect in zebrafish as edema which was indication for kidney function defect in zebrafish (Fig. 5g). Electron microscope confirmed the kidney injury as loss of tubular brush-border and increased the space between tubules (Fig. 5h). The edema ratio decreased with overexpression of human SLC34A1 mRNA (Fig. 5i).

**Fig. 6.**
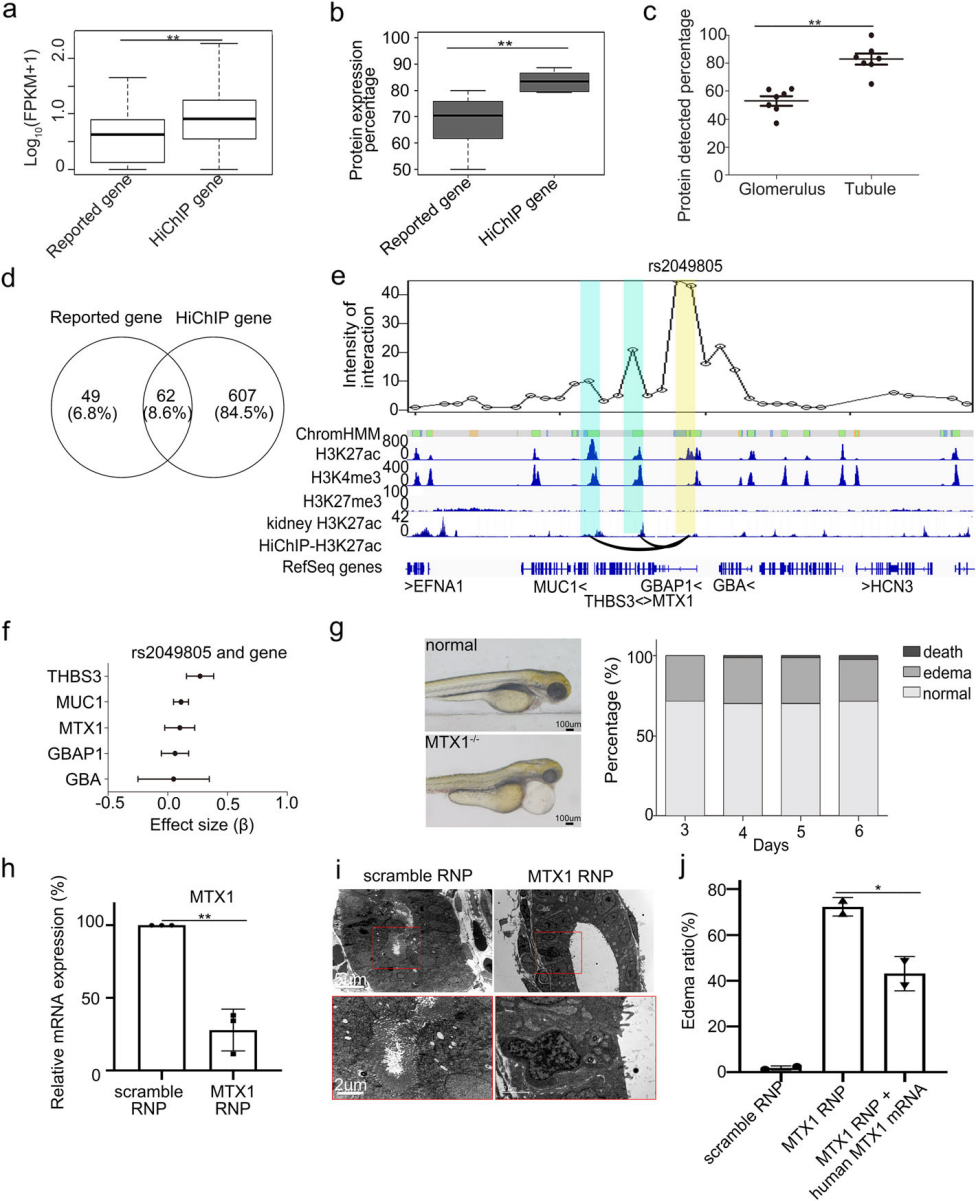
Identification of target genes for trait-associated SNPs overlapping with enhancers. **a** The transcription for reported and HiChIP genes in tubular cells. ** represents *p* value < 0.01. **b** The percentages of expressed genes in kidney tubules for reported and HiChIP genes based on protein levels from HPA. ** represent *p* value < 0.01. **c** The percentages of expressed genes for HiChIP genes in glomeruli or tubules based on protein levels from HPA. ** represents *p* value < 0.01. **d** Overlapping between HiChIP genes and reported genes. **e** Top: Virtual 4C of chromatin interaction around rs2049805. Bottom: epigenomic profile including ChromHMM, histone modifications in tubule cells, histone modification in kidney, chromatin interactions, and gene annotation track. Yellow bar highlights rs2049805 enhancer and anchor of virtual 4C. Blue bar highlights promoters interacted with rs2049805. **f** The effect size (*β*) of eQTL association between rs2049805 and THBS3, MUC1, MTX1, GBAP1, and GBA in tubulointerstitium. **g** Left: representative figures show edema of MTX1^−/−^ zebrafish. Right: edema ratio of MTX1^−/−^ zebrafish at different days. **h** qPCR of MTX1 transcription in zebrafish with RNP (sgRNA and CRISPR/Cas9 protein complex) of scramble, MTX1 sgRNA. Each group was repeated three times. **i** Electron microscope of kidney from zebrafish with scramble, MTX1 RNP. The red boxes (top) show increase space between tubules, and they are enlarged (bottom) to show the shed off of brush-border fragment. **j** Overexpression of human MTX1 mRNA partially rescue the edema. Each group was examined on two batches

In addition to functional SNPs at promoters, we also investigated SNPs overlapping with enhancers. We identified 669 target genes through our 3D network for 7 kidney-related traits (Additional file 5: Table S8 and Additional file 6: Table S9), including genes have been reported playing roles in CKD as CYP24A1 [31] or associated with RCC as CCND1 [32]. The expression of these identified HiChIP target genes was higher than reported genes at bulk transcription level in HK2 cells (Fig. 6a), at scRNA level in kidney proximal tubules (Additional file 1: Fig. S8) and at protein level in kidney tubule (Fig. 6b). As target genes associated with promoter SNPs, the target genes for enhancer SNPs also showed tubular biased expression, as with a significantly higher expression proportion in kidney tubule (81.9%) than in kidney glomerulus (56%) (Fig. 6c).

In addition to previously reported genes based on genomic proximity, using our pipeline, we identified 607 new genes as targets for disease-associated variants (Fig. 6d). For example, rs2049805 which associated with renal function-related traits (BUN) was identified as active enhancer in our landscape (Fig. 6e). The nearest gene of rs2049805 was GBAP1. We found three more genes THBS3, MTX1 and MUC1 were potential targets as their promoters interacted with the rs2049805 located enhancer. The role of rs2049805 on MUC1 has been reported and investigated previously [12, 33], confirming the accuracy of our discovery. By checking the eQTL profile, we found the eQTL effects of rs2049805 on both THBS3 and MTX1 were higher than that on GBAP1 (Fig. 6f). Both THBS3 and MTX1 were tubular-specifically expressed (Additional file 1: Fig. S9a), and the expressions were greatly downregulated in chronic kidney disease (Additional file 1: Fig. S9b). We then continued to examine the role of THBS3 and MTX1 by CRISPR/cas9 genomic editing in zebrafish. THBS3 knockout did not show phenotype change (data was not shown), probably because there was a compensatory effect between THBS homolog genes [34]. MTX1 knockout zebrafish showed kidney function injury (Fig. 6g–j). All these findings indicated that cell type-specific epigenetic landscape could contribute to the identification of cell type-specific functional variants and provided molecular annotation of the non-coding SNPs in disease association.

## Discussion

In this study, we integrated 1D and 3D epigenetic landscapes to leverage the annotation of disease-associated variants. Most of the GWAS hits locate at non-coding regulatory regions which play cell type-specific roles in genome function. For example, the obesity and type 2 diabetes-associated SNP rs9930506 is discovered to function as an enhancer only in brain [35], and the CKD-associated SNP rs11959928 is found as eQTL only in kidney tubule but not in glomeruli or the whole kidney [28]. Thus, it is of great significance to annotate GWAS hits in a cell type-specific way to understand the underlying mechanisms of the diseases. In this study, the epigenetic features in kidney tubule cells coordinated well with the specific expression pattern. Only genes with promoters marked by active epigenetic marks are highly expressed while promoters marked by repressive markers are not expressed at all. With this information, it is feasible to identify functional SNPs in certain tissue or cell type relevant for diseases. However, HK2 as a cell line representing kidney proximal tubule is not a perfect match of native kidney cells. The culture leads to loss of some biological features in vivo as endocrine functions [36]. The cell line seems to be suitable for study of some functions but not all the characteristics of proximal tubular cell. Further characterization of epigenetic landscape in native kidney cells is required for comprehensive investigation of the cell type-specific function of kidney disease risk SNPs.

eQTL analysis has been widely used to find associated genes for variants on the population level [37]. However, eQTL analysis requires a large sample size, and its application to a large amount of tissues and cell types is impeded. Epigenetic profiles generated on a few samples would provide enough information for cell type-specific genomic annotation. The two strategies can compensate for each other and produce valuable annotation of disease-associated variants. In this study, we identified target genes of rs2049805 based on one set of epigenetic landscape. The same result has been discovered by eQTL analysis in NephQTL [38], which is obtained using 166 samples. In addition to the advantage of reduced sample size, the epigenetic information as chromatin contact reveals the real regulatory target which interacts with each other physically, not only based on association relationships. This property would reduce the number of false positive in target gene identification. The high-resolution epigenetic analysis represents as an efficient and trustable tool for GWAS annotation.

Through the comprehensive epigenetic landscape, we can discover functional variant-target gene pairs. In this study, we identified risk-associated genes like SLC34A1 and MTX1. SLC34A1 is a sodium-phosphate transporter which is supposed to be important for the function of kidney tubules [39]. Loss-of-function mutation in protein-coding regions of SLC34A1 results in abnormal kidney function as renal Fanconi’s syndrome [40]. MTX1 is a mitochondrial outer membrane protein [41]. Loss of its homolog MTX2 leads to mitochondrial dysfunction [42]. The mammalian kidney tubule relies on abundant mitochondria to provide the energy required for constant reclamation [43]. Although the role of MTX1 in kidney is not clear yet, our CRISPR/cas9 editing zebrafish demonstrates MTX1 is indispensible for normal kidney function. Further investigation of the mechanism of these genes in kidney function would help us better understand the genetic risk of kidney disease.

In addition to SLC34A1 and MTX1, we also discovered genes which are indicated to be disease associated. CYP24A1, for example, is a novel target gene for rs17216707 and rs6127099 which associate with CKD. CYP24A1 is previously reported to regulate FGF-23 signaling and affected bone and mineral metabolism during the process of CKD [31]. For RCC, CCND1 has been discovered as a biomarker for clear cell renal cell carcinoma [32]. In this study, we found CCND1 is a target gene of rs4980785 and rs11263654 which are RCC-associated variants. This discovery provides interpretation to the potential role of SNPs rs4980785 and rs11263654 in RCC. Further investigation of these novel target genes and their regulatory SNPs would facilitate genetic detection of disease risk.

## Conclusions

We established a powerful pipeline to generate high-resolution epigenetic landscapes to functionally annotate GWAS hits in a cell type-specific way, and provided an informative resource for the functional annotation of genomic loci in kidney tubule cells. Most kidney diseases are complex diseases, which could be induced by alterations in various tissues or cell types. Application of this pipeline to other cell types in kidney with accumulation of epigenetic landscape in a broader range would provide more comprehensive understanding of kidney diseases.

## Methods

### Cell culture and treatment

Human kidney tubule epithelial cell culture HK2 cell line was purchased from ATCC and maintained in DMEM-F12 medium with 10% FBS (Gibco), 1% penicillin-streptomycin, and 0.05% DMSO at 37 °C in a 5% (v/v) CO_2_ humidified incubator.

### RNA-seq

Cells were harvested in Trizol (Invitrogen). The total RNA was enriched by depletion of rRNA. The library was constructed and sequenced by Vazyme Biotech Company.

### ChIP-seq

ChIP assays were performed as previously described [44]. In total, 0.5 million cells were used for each experiment. The chromatin was sheared on the Sonics VCX-130 with 15 s on and 30 s off for 12 cycles. Immunoprecipitation was performed using 3–5 μg of antibodies (H3K27ac, Abcam, ab4729; H3K27me3, diagenode, C15410069, H3K4me3, Abcam, ab8580). DNA was purified by DNA Clean & Concentrator-5 kit (ZYMO). Libraries were constructed as previously indicated [45], and sequenced on HiSeq4000.

### HiChIP

HiChIP was performed according to the published protocol [24], with the following modifications: 1 million cells were used for each sample; fixed and isolated nuclei were digested with MboI restriction endonuclease (NEB R0147S); after ligation, the nuclei with in situ-generated contacts were sheared on Sonics VCX-130 with 15 s on and 30s off for 4 cycles; the antibody to H3K27ac (Abcam, ab4729) was used for pull down. The samples were sequenced on an Illumina HiSeq4000 or used for qPCR.

### CRISPR deletion and interference

CRISPR/Cas9 method to generate deletion mutant in G0 zebrafish was performed as previously described [46]. The sgRNAs of SLC34A1, THBS3 and MTX1 were designed by ZiFiT Targeter (Version 4.2). The synthetic oligo strands of sgRNA were annealed, then constructed in the pDR274 vector and transcribed by T7 In Vitro Transcription Kit (TR101-01; Vazyme Biotech). Then, 1 μg/μl Cas9 protein (Novoprotein) and 800 ng/μl sgRNAs were mixed to generate Cas9 Ribonucleoprotein Complex (RNP) and incubated at 37 °C for 5 min, followed by microinjection into 1-cell stage embryos in the yolk. The sgRNAs were listed in Additional file 1: Table S4. Each sgRNA was examined with three replications.

Capped and poly(A) tailed mRNA transcription and rescue injection. Human MTX1 and SLC34A1 coding sequences were synthesized in TSINGKE Biological Technology, amplified, and cloned into pEASY T5 Blunt Zero kit (Transgene). The DNA templates were purified (DNA Clean and Concentrator-5, ZYMO Research) and in vitro transcribed (HiScribe T7 ARCA mRNA Kit, NEB). mRNA was purified using RNA Clean and Concentrator-25 (ZYMO Research). RNP and mRNA (25 ng/μl) mixture were microinjected into 1-cell stage embryos in the yolk. The primers for SLC34A1 are sense: 5′-ATGTTGTCCTACGGAGAGAGGC-3′, antisense: 5′-CTAGAGGCGGGTGGCATTG-3′, for MTX1 sense: 5′- ATGCTGCTCGGGGGACCC-3′, antisense: 5′-TCATTCCTCTTCATCCTCCTCAG − 3′. Each condition was tested on two batches, and each batch included over 100 zebrafish.

dCas9-KRAB-sgRNA plasmids were constructed by KeyGen BioTech. Each plasmid was transfected to HK2 cells by Lipo2000 (Invitrogen). Total RNA was extracted using Trizol reagent (Invitrogen). Real-time PCR data were calculated using the ∆∆CT method. Three replicates were carried out for each plasmid. sgRNAs were target for rs6420094 region 1 was 5′-TCCAGGGAAGCTATGCACCA-3′, and for region 2 was 5′-GGAGACGACTCCAGAGATAG-3′. Negative control, sgRNA1, and sgRNA2 were repeated three times.

### Transmission electron microscopy

Embryos were fixed in cold 3.75% glutaraldehyde for 12 h at 4 °C, and followed by standard procedure [47]. The specimens were examined and photographed with Hitachi 7500 TEM.

### Bioinformatics

#### RNA-seq analysis

RNA-seq analysis was performed as previously described [48]. The adaptors-trimmed and quality-filtered reads were mapped to hg19 using HISAT2 with default parameters. Transcript assembly was performed using Stringtie. Expression level estimation was reported as fragments per kilobase of transcript sequence per million mapped fragments (FPKM).

#### ChIP-seq analysis

The adaptors-trimmed and quality-filtered reads were aligned to the hg19 using Bowtie2 [49] with default parameters, and uniquely mapped reads were used for peak calling with MACS2 [50].

#### Chromatin segmentation and annotation

Chromatin was segmented and annotated using ChromHMM [51] based on three key histone modifications including H3K27ac, H3K27me3 and H3K4me3.

#### HiChIP analysis

HiChIP paired-end reads for each replication were aligned to hg19 genomes using the HiC-Pro pipeline [52] with default parameters to assign reads to MboI restriction fragments, filtered for valid pairs, and generated binned interaction matrices. The valid pairs for replicates were then combined for loop calling with Hichipper [53]. Chromatin interaction heatmap was generated with Juicebox [54] on valid pairs. Virtual 4C data was extracted with the Juicebox tool “dump.” The promoter-promoter, promoter-enhancer, and enhancer-enhancer interactions were annotated on Hichipper called interactions. Promoters were defined as 2 kb around RefSeq TSS of protein-coding. Enhancers were defined as active enhancer identified by ChromHMM.

#### GWAS analysis

GWAS SNPs were extracted from GWAS Catalog (downloaded 30 September 2018; https://www.ebi.ac.uk/gwas/). We chose non-kidney-related traits the same as Farh et al. [3]. Kidney-related traits with more than 15 known trait-associated SNPs were kept. CKD SNPs were collected from GWAS Catalog overlapping with CKDGen Consortium studies (downloaded 24 April 2019; http://ckdgen.imbi.uni-freiburg.de/) not on diabetes. The coordinations of SNPs were lift over to hg19 with UCSC tools.

GWAS hit bootstrap enrichment analysis was performed as follows. For each trait, the numbers of SNPs were counted as “*N*_trait_.” The SNPs were then intersected with one chromatin category and the number of overlapped SNPs was “*N*_trait-o_.” We then randomly selected “*N*_trait_” SNPs from the total SNP pool of all traits and the number of these SNPs overlapping the same category was “*N*_radom-o_.” The procedures were repeated 5000 times to obtain the distribution of *N*_radom-o_ which was a normal distribution. The *p* vaule of *N*_trait_ was calculated based on the normal distribution of *N*_radom-o_ with pnorm in *R*.

#### Other data resource

eQTL information was obtained from NephQTL [55] (http://nephqtl.org/searchResult). The 95% confidence interval was calculated as *beta*±*beta*/*t*-statistics*t_0.975 _(here *t*0.975 = 1.974).

scRNA-seq of human kidneys were from three studies [56–58]. The gene expression matrix was generated in Seurat with parameters provided in the reference or default parameters. Genes with average expression in proximal tubular cell type greater than 0 were characterized as expressed genes.

Gene expression at protein level for tubule or glomeruli was obtained from Human Protein atlas [59] (https://www.proteinatlas.org/) or Nephroseq (https://www.nephroseq.org).

### Statistics

The following statistical tests were performed or otherwise described in bioinformatics analysis: two-tailed Student *t* test (Fig. 2a, Fig. 2c, Fig. 2e, Fig. 3d, Fig. 3e, Fig. 5e, Fig. 5f, Fig. 6a, Fig. 6b, Fig. 6c, Fig. 6h, Fig. S5b), Fisher’s exact test (Fig. 5i and Fig. 6j), and pnorm in *R* (Fig. 4b, Fig. S6).

## Supplementary Information

**Additional file 1: Fig. **Correlation of replications for histione modifications ChIP-seq. **Fig. S2.** Histone modifications and RNA tracks for HK2 cell line and whole kidney. **Fig. S3.** Histone modification tracks at DAB2 enhancer. **Fig. S4.** Immunochemistry of NUDT7, VAT1L, CLEC3A and WWOX in kidney tubule from Protein atlas. **Fig. S5.** Quality control of H3K27ac-HiChIP. **Fig. S6.** Enrichment of GWAS hits in regulatory elements. **Fig. S7.** Expression of SLC34A1 in human kidney. **Fig. S8.** Expression of HiChIP genes and reported genes in scRNA-seq. **Fig. S9.** Expression levels of THBS3 and MTX1 in human kidney. **Table S1.** Statistics of ChIP-seq. **Table S2.** Statistics of HiChIP. **Table S3.** Numbers of GWAS SNPs overlapping with regulatory elements for kidney related traits. **Table S4.** sgRNAs for CRISPR/Cas9.

**Additional file 2: Table S5.** Enhancers which located at the SNPs of kidney diseases distributed in different tissues.

**Additional file 3: Table S6.** Identified target genes for lead SNPs of kidney related traits GWAS overlapping with promoters.

**Additional file 4: Table S7.** Identified target genes for ldSNPs of kidney related traits GWAS overlapping with promoters.

**Additional file 5: Table S8.** Identified target genes for lead SNPs of kidney related traits GWAS overlapping with enhancers.

**Additional file 6: Table S9.** Identified target genes for ldSNPs of kidney related traits GWAS overlapping with enhancers.

**Additional file 7.** The individual data values used in the figure.

## Data Availability

All data generated or analyzed during this study are included in this published article, its supplementary information files and publicly available repositories. RNA-seq, ChIP-seq, and HiChIP-seq data used in this study have been deposited in the GEO database under the accession codes GSE147646 [60]. For figures with *n* less than 6, all individual data values are provided in Additional file 7.

## Abbreviations

GWAS: genome-wide association studies
REMC: Roadmap Epigenome Mapping Consortium
4C-seq: circular chromatin conformation capture
Hi-C: High-throughput chromosome conformation capture
HiChIP: In situ Hi-C followed by chromatin immunoprecipitation
RCC: Renal cell carcinoma
CKD: Chronic kidney disease
eQTL: Expression quantitative trait loci
