# Supplementary material for: Chromatin architecture reveals cell type-specific target genes for kidney disease risk variants
Source: BMC Biol. 2021 Feb 24;19:38. doi: 10.1186/s12915-021-00977-7 (PMC7905576; doi:10.1186/s12915-021-00977-7)
Supplement: Supplementary file 1 — Additional file 1: Fig. Correlation of replications for histione modifications ChIP-seq. Fig. S2. Histone modifications and RNA tracks for HK2 cell line and whole kidney. Fig. S3. Histone modification tracks at DAB2 enhancer. Fig. S4. Immunochemistry of NUDT7, VAT1L, CLEC3A and WWOX in kidney tubule from Protein atlas. Fig. S5. Quality control of H3K27ac-HiChIP. Fig. S6. Enrichment of GWAS hits in regulatory elements. Fig. S7. Expression of SLC34A1 in human kidney. Fig. S8. Expression of HiChIP genes and reported genes in scRNA-seq. Fig. S9. Expression levels of THBS3 and MTX1 in human kidney. Table S1. Statistics of ChIP-seq. Table S2. Statistics of HiChIP. Table S3. Numbers of GWAS SNPs overlapping with regulatory elements for kidney related traits. Table S4. sgRNAs for CRISPR/Cas9. [file 12915_2021_977_MOESM1_ESM.docx]

Additional file 1:

**Fig. S1** Correlation of replications for histione modifications ChIP-seq.

**Fig. S2** Histone modifications and RNA tracks for HK2 cell line and whole kidney.

**Fig. S3** Histone modification tracks at DAB2 enhancer.

**Fig. S4** Immunochemistry of NUDT7, VAT1L, CLEC3A and WWOX in kidney tubule from Protein atlas.

**Fig. S5** Quality control of H3K27ac-HiChIP.

**Fig. S6** Enrichment of GWAS hits in regulatory elements.

**Fig. S7** Expression of SLC34A1 in human kidney.

**Fig. S8** Expression of HiChIP genes and reported genes in scRNA-seq.

**Fig. S9** Expression levels of THBS3 and MTX1 in human kidney.

**Table S1** Statistics of ChIP-seq.

**Table S2** Statistics of HiChIP.

**Table S3** Numbers of GWAS SNPs overlapping with regulatory elements for kidney related traits.

**Table S4** sgRNAs for CRISPR/Cas9.


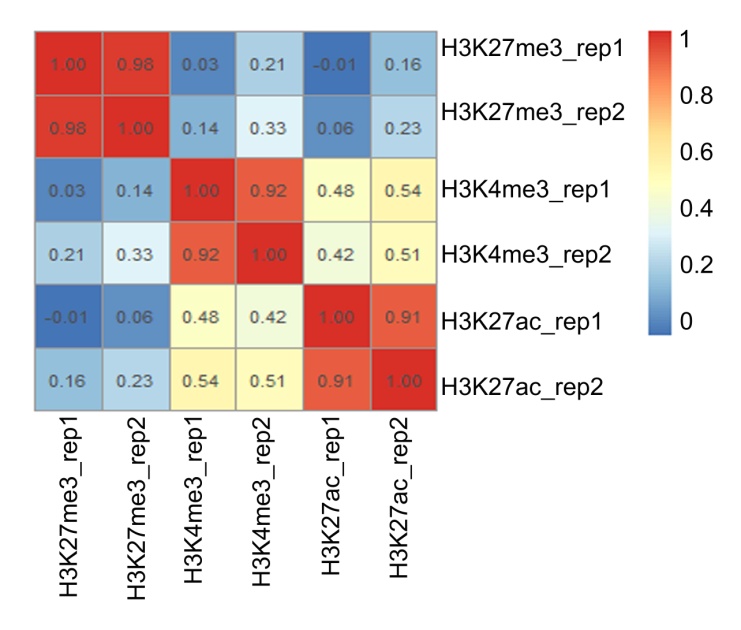


**Fig. S1** Correlation of replications for histione modifications ChIP-seq.

**
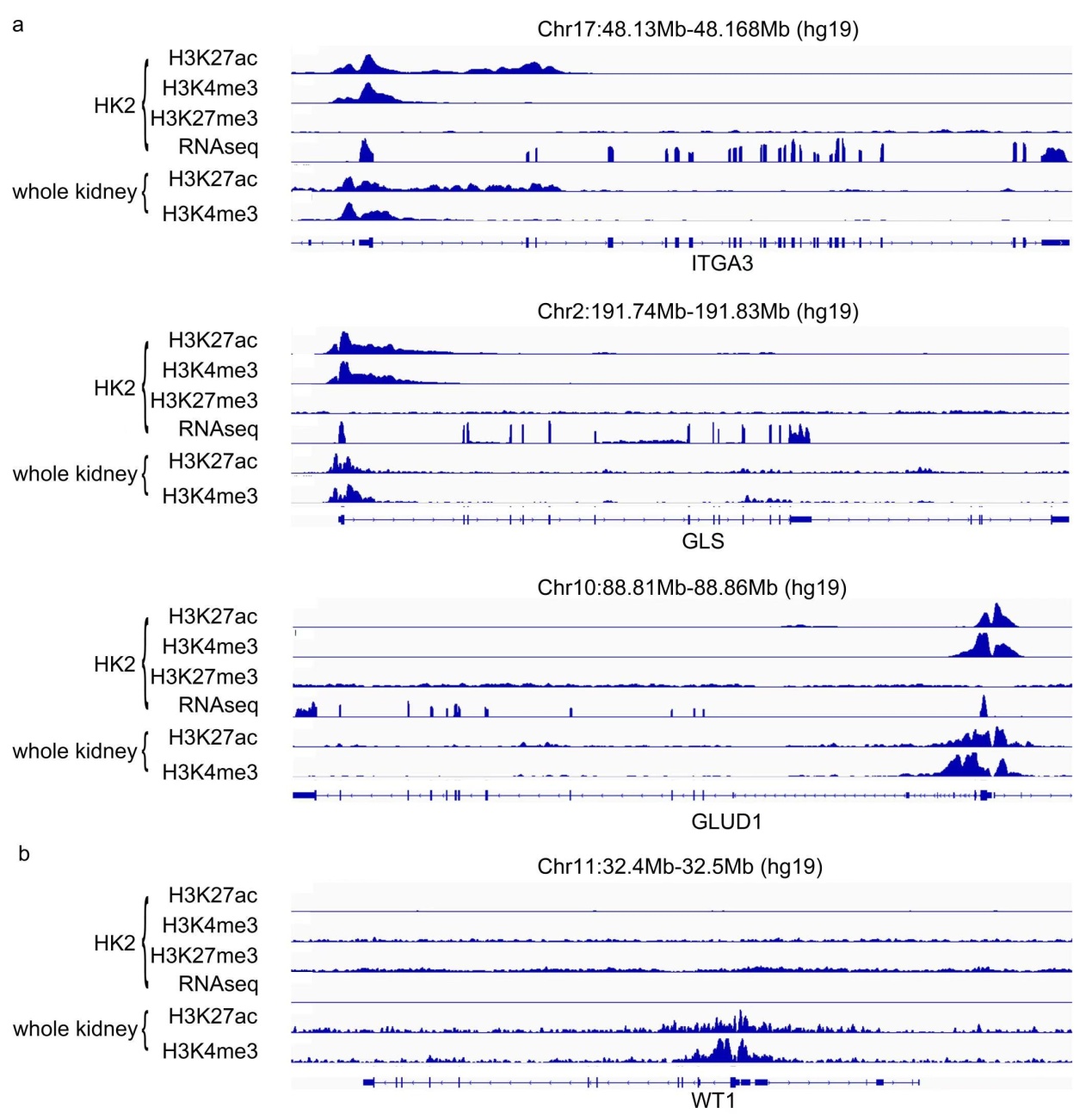
Fig. S2** Histone modifications and RNA tracks for HK2 cell line and whole kidney. (a) Histone modification and RNA tracks for tubular feature genes ITGA3, GLS and GLUD1. (b) Histone modification and RNA tracks for glomeruli feature genes WT1.


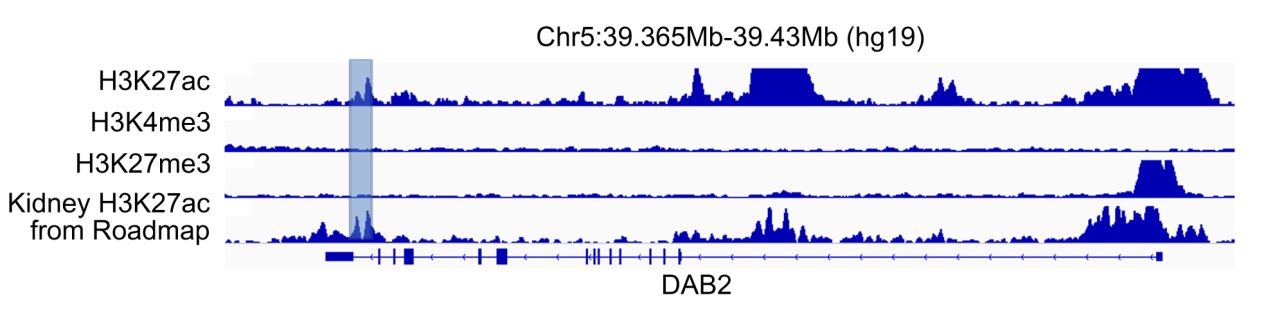


**Fig. S3** Histone modification tracks at DAB2 enhancer. Blue block highlights the location of the enhancer.


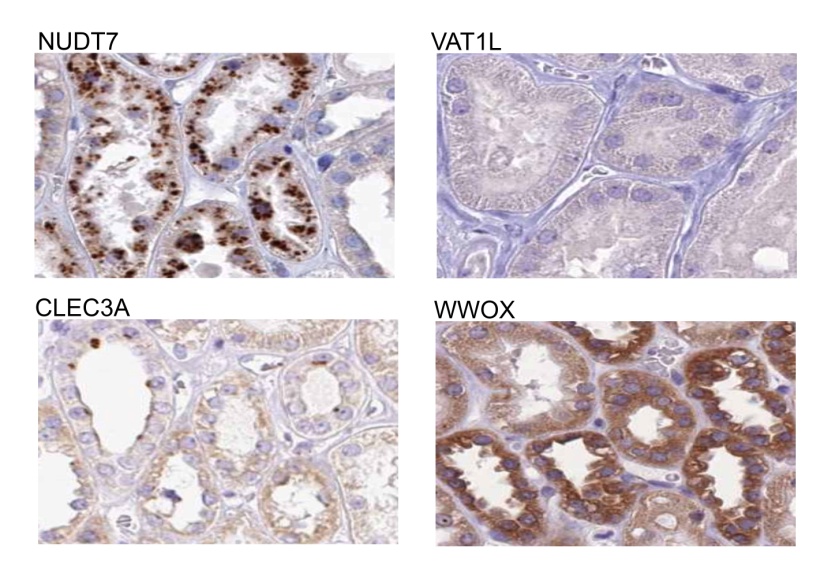


**Fig. S4** Immunochemistry of NUDT7, VAT1L, CLEC3A and WWOX in kidney tubule from Protein atlas.


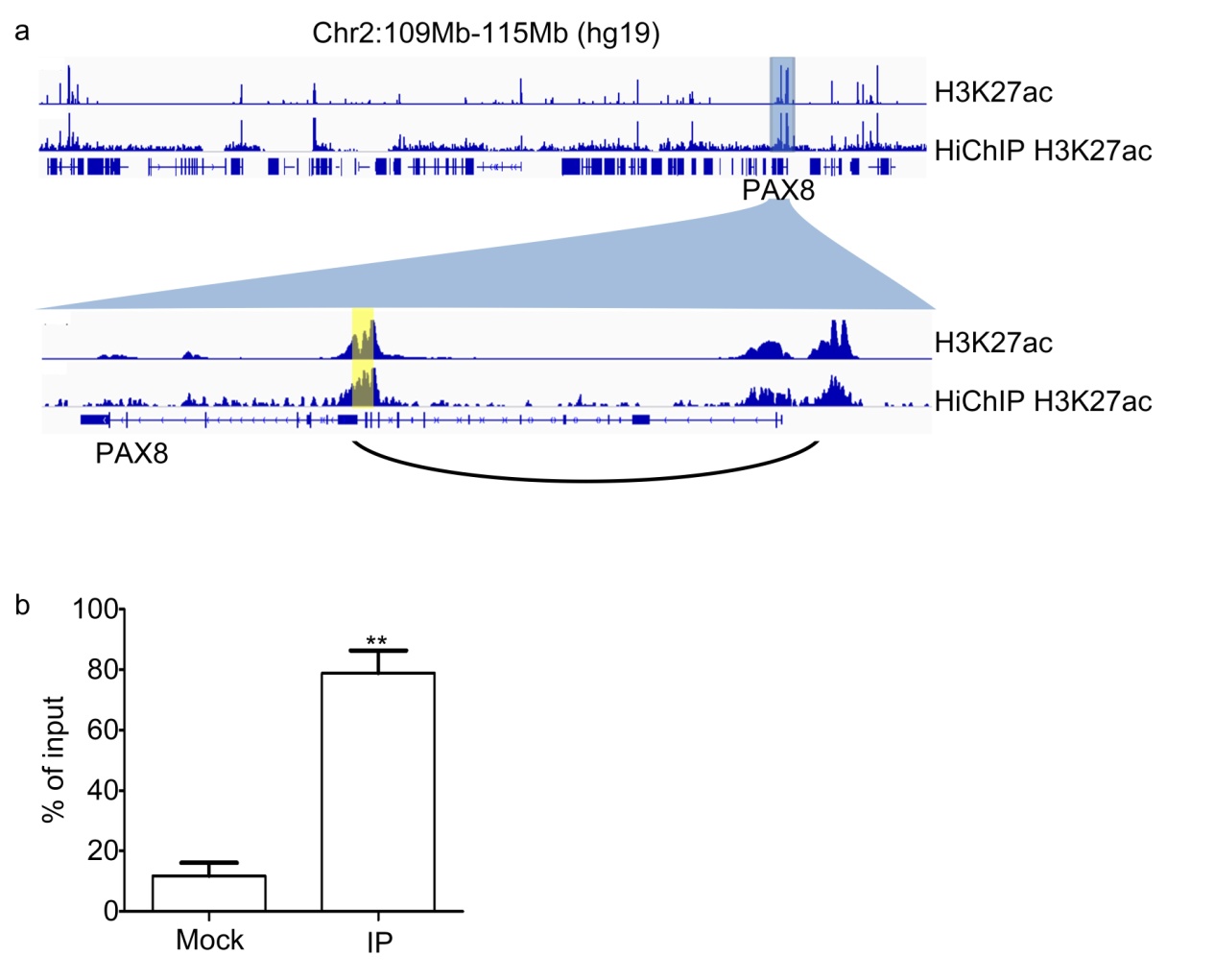


**Fig. S5** Quality control of H3K27ac-HiChIP. (a) Tracks for H3K27ac ChIP-seq results and H3K27ac-HiChIP 1D signal. The blue block highlights the region which is enlarged at the bottom. Yellow block highlights the anchor which is tested in b. (b) ChIP-qPCR of H3K27ac-HiChIP sample at an anchor in PAX8 loci (highlighted in yellow block in (a). Mock: without H3K27ac antibody; IP: with H3K27ac antibody. n = 3.

**
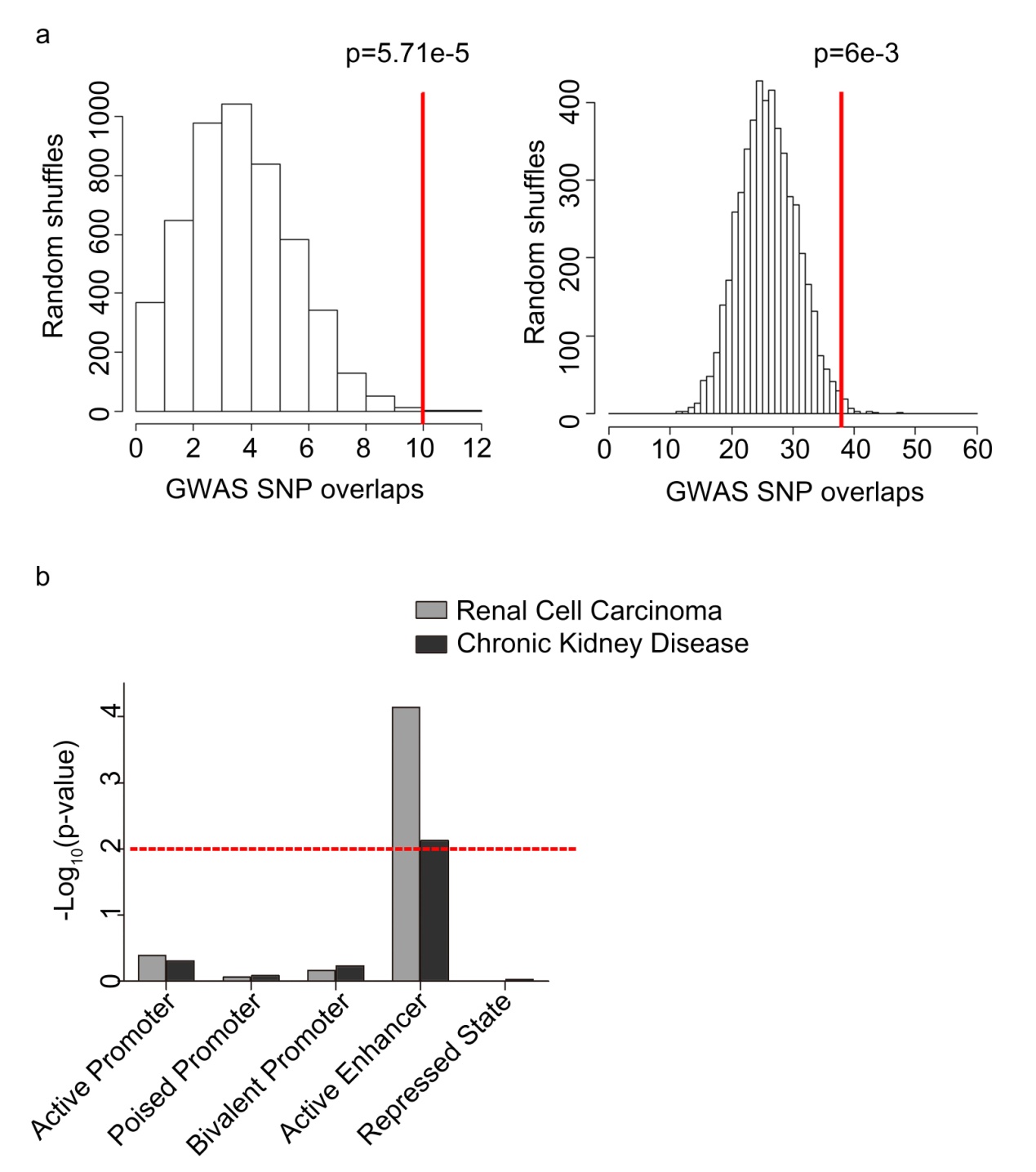
Fig. S6** Enrichment of GWAS hits in regulatory elements. (a) The distribution of the numbers of randomly selected SNPs overlapping with active enhancers in HK2 cells. Red lines highlight the number of disease associated SNPs overlapping active enhancer for renal cell carcinoma (left) and chronic kidney disease (right). (b) The enrichment of disease associated SNPs on different types of regulatory elements in HK2 cells. Red dash line highlights p-value=0.01.

**
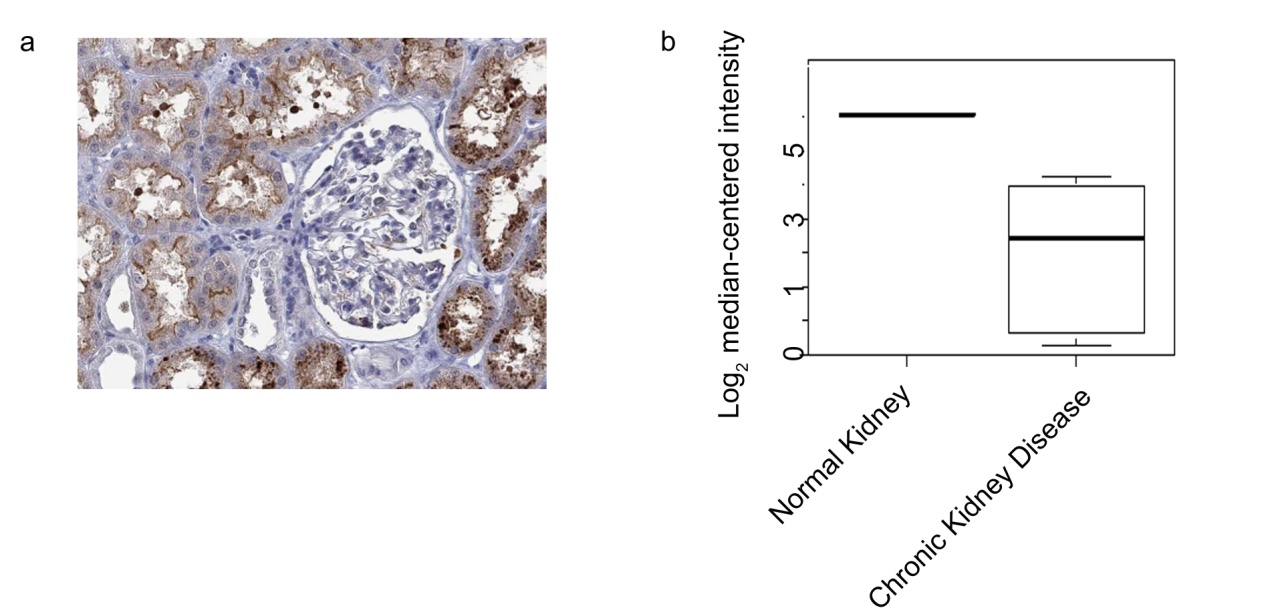
Fig. S7** Expression of SLC34A1 in human kidney. (a) Immunochemistry of SLC34A1 shows strong expression in kidney tubules (from Human Protein Atlas). (b) Transcription of SLC34A1 in kidney for normal and chronic kidney disease biopsy (from Nephroseq). n (Normal Kidney) =3, n (Chronic Kidney Disease) =5.

**
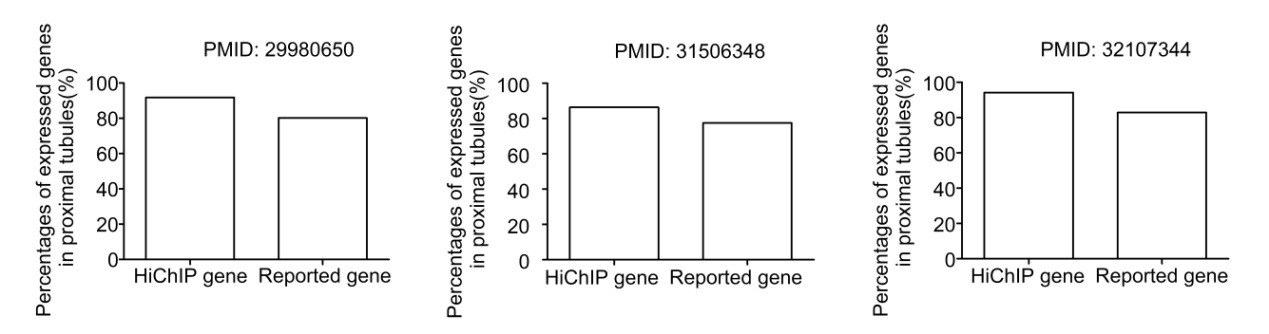
Fig. S8** SLC34A1 and MTX1 are required for kidney function in zebrafish. HiChIP genes express more often than reported genes in proximal tubular cell in three kidney scRNA-seq studies.


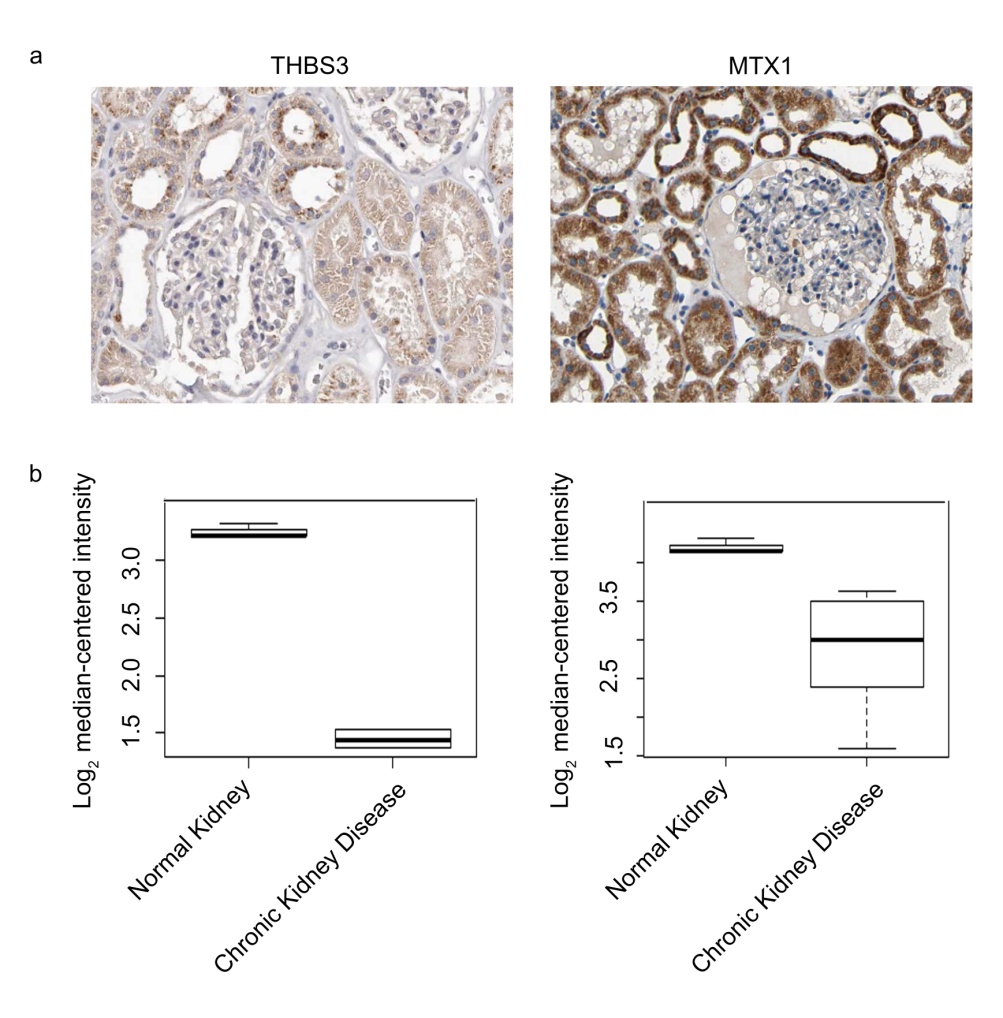


**Fig. S9** Expression levels of THBS3 and MTX1 in human kidney. (a) The protein expression of THBS3, and MTX1 in kidney from Protein atlas. (b) Transcription of THBS3, and MTX1 in kidney for normal and chronic kidney disease from Nephroseq in Nakagawa CKD Kidney study validation set group. n (Normal Kidney) =3, n (Chronic Kidney Disease) =5.

**Table S1** Statistics of ChIP-seq.

Sequence depth and FRiP of histone ChIP-seq. FRiP: fraction of all mapped reads that fall into peak regions.

|  | Total read pairs | Aligned read pairs | FRiP |
| --- | --- | --- | --- |
| H3K27ac_rep1 | 57019697 | 40550340 | 0.769 |
| H3K27ac_rep2 | 47884317 | 37398311 | 0.133 |
| H3K27me3_rep1 | 81366771 | 63371212 | 0.05 |
| H3K27me3_rep2 | 65143476 | 37857952 | 0.054 |
| H3K4me3_rep1 | 71010708 | 57519130 | 0.328 |
| H3K4me3_rep2 | 40562489 | 25656120 | 0.186 |

**Table S2** Statistics of HiChIP.

|  | HiChIP_H3K27ac_rep1 | | HiChIP_H3K27ac_rep2 | |
| --- | --- | --- | --- | --- |
| Total_pairs_processed | 249979214 | 100% | 283114204 | 100% |
| Unmapped_pairs | 7170057 | 2.87% | 7453364 | 2.63% |
| Low_qual_pairs | 0 | 0 | 0 | 0 |
| Unique_paired_alignments | 183414094 | 73.37% | 204920622 | 72.38% |
| Multiple_pairs_alignments | 28741720 | 11.50% | 31045065 | 10.97% |
| Pairs_with_singleton | 30653343 | 12.26% | 39695153 | 14.02% |
| Low_qual_singleton | 0 | 0 | 0 | 0 |
| Unique_singleton_alignments | 0 | 0 | 0 | 0 |
| Multiple_singleton_alignments | 0 | 0 | 0 | 0 |
| Reported_pairs | 183414094 | 73.37% | 204920622 | 7238.10% |
| Valid_interaction_pairs | 154941557 | 61.98% | 168353100 | 59.46% |
| Valid_interaction_pairs_FF | 38442470 | 15.38% | 41685847 | 14.72% |
| Valid_interaction_pairs_RR | 38626935 | 15.45% | 41812788 | 14.77% |
| Valid_interaction_pairs_RF | 38639290 | 15.46% | 41557148 | 14.68% |
| Valid_interaction_pairs_FR | 39232862 | 15.69% | 43297317 | 15.29% |
| Dangling_end_pairs | 6500437 | 2.60% | 10240472 | 3.62% |
| Religation_pairs | 2565695 | 1.03% | 4531026 | 1.60% |
| Self_Cycle_pairs | 210822 | 0.08% | 214651 | 0.08% |
| Single-end_pairs | 0 | 0.00% | 0 | 0.00% |
| Dumped_pairs | 19195583 | 7.68% | 21581373 | 7.62% |

**Table S3** Numbers of GWAS SNPs overlapping with regulatory elements for kidney related traits.

|  | active promoter | poised promoter | bivalent promoter | active enhancer | repressed state |
| --- | --- | --- | --- | --- | --- |
| Chronic kidney disease | 12  (8.57%) | 4  (2.86%) | 1  (0.71%) | 26  (18.57%) | 26  (18.57%) |
| Diabetic kidney disease | 0  (0.00%) | 0  (0.00%) | 0  (0.00%) | 6  (11.11%) | 10  (18.52%) |
| Idiopathic membranous nephropathy | 4  (19.05%) | 3  (14.29%) | 0  (0.00%) | 3  (14.29%) | 8  (38.10%) |
| IgA nephropathy | 5  (15.15%) | 2  (6.06%) | 0  (0.00%) | 5  (15.15%) | 11  (33.33%) |
| Lupus nephritis in systemic lupus erythematosus | 1  (5.88%) | 0  (0.00%) | 0  (0.00%) | 1  (5.88%) | 4  (23.53%) |
| Renal cell carcinoma | 2  (6.25%) | 0  (0.00%) | 0  (0.00%) | 10  (31.25%) | 2  (6.25%) |
| Renal function-related traits (BUN) | 2  (13.33%) | 2  (13.33%) | 0  (0.00%) | 2  (13.33%) | 5  (33.33%) |

**Table S4** sgRNAs for CRISPR/Cas9.

| SLC34A1_sgRNA_1 | TAATACGACTCACTATAGGAGTTGAAACGCGGAGCTTGTTTTAGAGCTAGAAATAGC |
| --- | --- |
| SLC34A1_sgRNA_2 | TAATACGACTCACTATAGGCTGGGCGACGAGTCCATGGTTTTAGAGCTAGAAATAGC |
| SLC34A1_sgRNA_3 | TAATACGACTCACTATAGGTGACCCCTTCGATGACCTGTTTTAGAGCTAGAAATAGC |
| SLC34A1_sgRNA_4 | TAATACGACTCACTATAGGTGTGTACCTGGCAGGCGGGTTTTAGAGCTAGAAATAGC |
| THBS3_sgRNA_1 | TAATACGACTCACTATAGGGTGTCGGAGGCCTGCGCCGTTTTAGAGCTAGAAATAGC |
| THBS3_sgRNA_2 | TAATACGACTCACTATAGGTCGGTGAGAGCGCCAGCCGTTTTAGAGCTAGAAATAGC |
| THBS3_sgRNA_3 | TAATACGACTCACTATAGGAGTGAGCGTTCGTGTCACGTTTTAGAGCTAGAAATAGC |
| THBS3_sgRNA_4 | TAATACGACTCACTATAGGTAGACAATGACTTAGTCGGTTTTAGAGCTAGAAATAGC |
| MTX1_sgRNA_1 | TAATACGACTCACTATAGGGCAAAGCGAGCGTATGCCGTTTTAGAGCTAGAAATAGC |
| MTX1_sgRNA_2 | TAATACGACTCACTATAGGGTGGAAGTGACACGCCGCGTTTTAGAGCTAGAAATAGC |
| MTX1_sgRNA_3 | TAATACGACTCACTATAGGGCGACTCCGACTGATCCGGTTTTAGAGCTAGAAATAGC |
| MTX1_sgRNA_4 | TAATACGACTCACTATAGGCAGAGGGCGTCAGTCGTCGTTTTAGAGCTAGAAATAGC |
